# Long-term retrospective observation study to evaluate effects of adiponectin on skeletal muscle in renal transplant recipients

**DOI:** 10.1038/s41598-020-67711-1

**Published:** 2020-07-01

**Authors:** Hiroki Adachi, Keiji Fujimoto, Ai Fujii, Keita Yamasaki, Keiichiro Okada, Toshikazu Matsuura, Kazuaki Okino, Kengo Furuichi, Hitoshi Yokoyama

**Affiliations:** Department of Nephrology, Kanazawa Medical University School of Medicine, 1-1 Daigaku, Uchinada, Ishikawa 920-0293 Japan

**Keywords:** Renal replacement therapy, Predictive markers, Skeletal muscle

## Abstract

Although it has been reported that chronic kidney disease exacerbates sarcopenia progression, the mechanisms of the process remain unclear. Fifty-one patients who underwent renal transplantation at our hospital since 1998 (31 males and 20 females; aged 29–52 years at the time of transplantation) were retrospectively examined for the relationships among the psoas muscle index (PMI), intramuscular adipose tissue content (IMAC), serum adiponectin fractions (high-/low-molecular-weight) and new-onset diabetes after transplantation (NODAT). Before transplantation, age at kidney transplantation negatively correlated with PMI and positively correlated with IMAC (rS = − 0.427, p < 0.01; rS = 0.464, p < 0.01, respectively). Both at 1 and 5 years after transplantation, PMI was higher than before transplantation (p < 0.01). IMAC transiently decreased to − 0.39 at 1 year after kidney transplantation but subsequently increased to − 0.36 at 5 years after kidney transplantation. Multivariate analyses revealed that the mean increase in high-molecular weight adiponectin concentrations was an exacerbating factor for the mean change in PMI (p = 0.003). Moreover, the mean increases in IMAC were exacerbating factors for NODAT. In conclusion, the increase in the PMI is associated with high–molecular weight adiponectin levels after renal transplantation.

## Introduction

Sarcopenia is defined as loss of the mass, strength, and function of skeletal muscles^1^. It is associated with not only a decline in the activities of daily living and falls, which results in the need for long-term care, but also with an increase in mortality and is a significant clinical and social problem in the developed countries^2^. Sarcopenia is classified into primary sarcopenia and secondary sarcopenia^3^. Primary sarcopenia is an age-associated decrease in muscle mass, whereas secondary sarcopenia is a decrease in muscle mass associated with a reduced activity level, malnutrition, organ failure, invasion and diseases, including cancer. Therefore, contributing factors for secondary sarcopenia should be considered in the same way as ageing. Metabolic acidosis and the increased expression of angiotensin II have been shown to decrease muscle mass in patients with chronic kidney disease, particularly patients on dialysis^4,5^. However, studies on sarcopenia in patients with kidney transplantation are very few. Although sarcopenia is a clinically important manifestation, it is difficult to objectively evaluate its progression. As the quantity and quality of skeletal muscle is an essential component for sarcopenia, we focused on psoas muscle images on computed tomography (CT). Hamaguchi et al. reported that a decrease in muscle mass, expressed as the psoas muscle index (PMI), was related to the mortality rate after transplantation^6^. Muscle quality has recently been attracting attention, and the fatty degeneration of muscle has been associated with ageing and muscle weakness^7^ as well as with the development of diabetes mellitus^8^. Kitajima et al. evaluated the fatty generation of muscle according to the intramuscular adipose tissue content (IMAC) by abdominal CT^9^. Therefore, we used PMI and IMAC as sarcopenia markers in this study.

A wide variety of cytokines, called myokines, are secreted from skeletal muscle cells. Adiponectin (ADPN) is a member of myokines and is also secreted from fat cells. There are complex regulating mechanisms involved in ADPN secretion and crosstalk with effector cells. ADPN exerts anti-atherosclerotic, anti-diabetic and anti-inflammatory effects^10^ and also plays some roles in muscle fibre remodelling and fat cell metabolisms. Moreover, it is reported that ADPN expression levels are decreased under the condition of increasing inflammatory cytokines, such as TNF-α and IL-6^11^. Therefore, crosstalk between muscle and fat cells by ADPN is speculated as a possible pathogenic mechanism of sarcopenia^12^. In addition, our previous study indicated that there was a strong negative correlation between serum high-molecular weight (HMW) ADPN concentrations and renal function^13^. Therefore, the fraction of ADPN is an important factor in these analyses. In addition to inflammatory processes, ADPN may contribute in cellular metabolism. It also plays a role in the onset of diabetes. ADPN increases the number of intramuscular mitochondria and enhances their function in skeletal muscles through ADPN receptor 1 signalling pathways^14^. In kidney transplantation, ischaemic stress in kidney induces various kinds of inflammatory processes. Additionally, steroid is one of the key immunosuppressive drugs for kidney transplantation, but it affects new onset diabetes. Although some ADPN fractions are speculated to play critical roles in the onset or progression of sarcopenia and newly onset diabetes, detailed studies on ADPN and sarcopenia in kidney transplantation are few.

In this study, we examined muscle mass and fatty degeneration as sarcopenia markers and evaluated relationships between these factors and serum ADPN levels. We found that HMW-ADPN levels indicate increase of muscle mass until 5 years after kidney transplantation.

## Results

### Baseline characteristics of patients classified according to sexes

The clinical characteristics of 31 male and 20 female patients at the beginning of observations (before transplantation) are shown in Table 1. Although no difference was observed in age between the gender at transplantation or duration of dialysis, body mass index (BMI) and serum albumin was higher in males. Systolic and diastolic blood pressure were controlled. No difference was observed in the total cholesterol (T Chol), TG or non-high-density lipoprotein cholesterol (non-HDL-C) levels between the gender. IMAC for assessing muscle fat change wash higher in female (p < 0.01) and PMI was higher in males (p < 0.01). No significant difference in gender was observed in medication both before and after transplantation.

**Table 1:** Baseline characteristics of study subjects classified according to gender.

Variable	Total	Male	Female
N	51	31	20
Age at transplantation (month)	41.0 (29.0, 52.0)	44.0 (29.3, 54.8)	33.5 (29.0, 44.5)
Duration of dialysis (month)	34.0 (10.3, 109.5)	40.0 (13.0, 164.3)	20.5 (9.0, 50.5)
BMI (kg/m^2^)	20.4 (18.7, 22.2)	21.5 (19.2, 23.5)	18.9 (17.7, 20.3)*
**Blood pressure (mmHg)**			
Systolic	138 (122, 145)	138 (122, 143)	135 (120, 148)
Diastolic	78 (72, 88)	78 (70, 88)	80 (73, 87)
Serum creatinine (mg/dL)	12.2 (9.7, 14.6)	13.6 (10.2, 15.3)	10.6 (7.9, 12.3)
Serum Ca (mg/dL)	9.3 (8.9, 9.7)	9.3 (8.8, 9.8)	9.3 (8.9, 9.6)
Serum albumin	4.0 (3.7, 4.1)	4.0 (3.9, 4.2)	3.7 (3.4, 4.1)*
Total cholesterol (mg/dL)	156.0 (135.8, 191.3)	156.0 (119.5, 188.5)	160.0 (143.5, 200.0)
HDL-C (mg/dL)	43.0 (33.0, 55.5)	40.0 (32.0, 52.8)	46.5 (42.0, 57.0)
Non-HDL-C (mg/dL)	115.0 (83.3, 138.5)	115.0 (82.3, 136.0)	113.0 (84.5, 152.5)
Blood sugar (mg/dL)	98.0 (86.1, 109.2)	99.0 (86.8, 119.0)	92.5 (83.5, 98.0)
PMI (cm^2^/m^2^)	3.03 (2.64, 4.00)	3.55 (2.93, 4.19)	2.62 (2.43, 3.21)**
IMAC	− 0.37 (− 0.42, − 0.29)	− 0.39 (− 0.48, − 0.33)	− 0.30 (− 0.40, − 0.21)**
**Therapeutic agent (drug use, %)**			
Steroids	51 (100%)	31 (100%)	20 (100%)
Anti-metabolites	51 (100%)	31 (100%)	20 (100%)
Calcineurin inhibitors	51 (100%)	31 (100%)	20 (100%)
Anti-hypertensive drugs	34 (66.7%)	26 (83.9%)	8 (40%)**
Statin	11 (21.5%)	7 (22.6%)	4 (20%)

Data are shown as median (IQR) values.

*BMI* body mass index, *HDL-C* high-density lipoprotein cholesterol, *PMI* psoas muscle mass index, *IMAC* intramuscular adipose tissue content.

*< 0.05, **< 0.01, male vs. female according to the Mann–Whitney test.

### Relationships between muscle imaging data (PMI and IMAC) and clinical factors (duration of dialysis and age at transplantation) before transplantation

**Figure 1:**
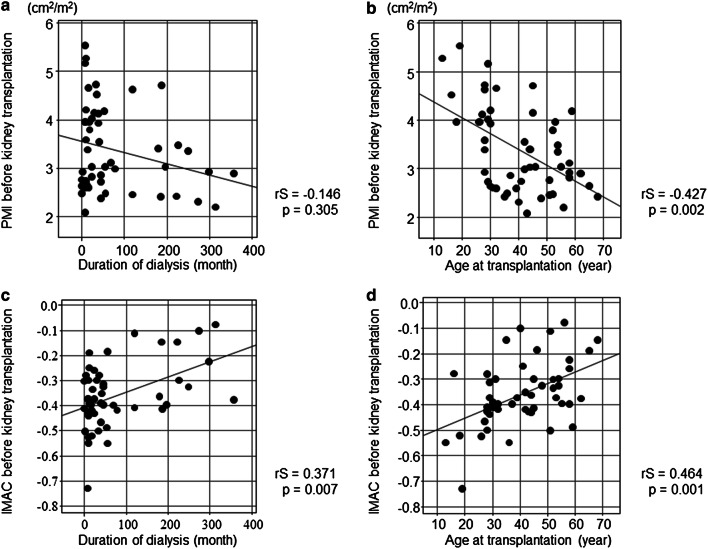
Relationships between PMI and IMAC before kidney transplantation, duration of dialysis and age at transplantation. (**a**) PMI before kidney transplantation did not correlate with the duration of dialysis (rS = − 0.146, p = 0.305, according to Spearman’s rank coefficient). (**b**) PMI before kidney transplantation negatively correlated with age at transplantation (rS =  − 0.427, p = 0.002, according to Spearman’s rank coefficient). (**c**, **d**) IMAC before kidney transplantation positively correlated with the duration of dialysis and age at kidney transplantation (rS = 0.371, p = 0.007 and rS = 0.464, p = 0.001, respectively, according to Spearman’s rank coefficient). *PMI* psoas muscle mass index, *IMAC* intramuscular adipose tissue content.

The relationships between muscle imaging data (PMI and IMAC) and clinical factors (duration of dialysis and age at transplantation) are shown in Fig. 1. PMI before transplantation did not correlate with the duration of dialysis but negatively correlated with age at transplantation (rS = − 0.427, p < 0.01). IMAC before transplantation positively correlated with the duration of dialysis and age at transplantation (rS = 0.371, p < 0.01 and rS = 0.464, p < 0.01, respectively). Factors that affected PMI and IMAC before transplantation were evaluated using multiple regression analysis (Table 2). Gender female and old age at the time of transplantation were identified as factors contributing to low PMI before transplantation, and Gender female, long-term dialysis and old age at the time of transplantation were identified as factors for high IMAC.

**Table 2:** Factors influencing PMI and IMAC before kidney transplantation.

	β	SE	Stdβ	t	p
**Objective variable: PMI before kidney transplantation**					
(Constant)	6.595	0.389			
Gender (male = 0, female = 1)	− 1.089	0.169	− 0.604	6.420	< 0.001
Age at transplantation (years)	− 0.041	0.006	− 0.640	6.806	< 0.001
**Objective variable: IMAC before kidney transplantation**					
(Constant)	− 0.766	0.057			
Gender (male = 0, female = 1)	0.143	0.024	0.557	5.843	< 0.001
Age at transplantation (years)	0.004	0.001	0.465	4.280	< 0.001
Duration of dialysis (month)	0.0004	0.0001	0.303	2.846	0.006

Explanatory variables: Sex, Age at transplantation, Duration of dialysis, HMW-ADPN, *BMI*, and non-HDL-C and HDL before kidney transplantation.

*PMI* psoas muscle mass index, *IMAC* intramuscular adipose tissue content.

### Changes in PMI and IMAC and after kidney transplantation

**Figure 2:**
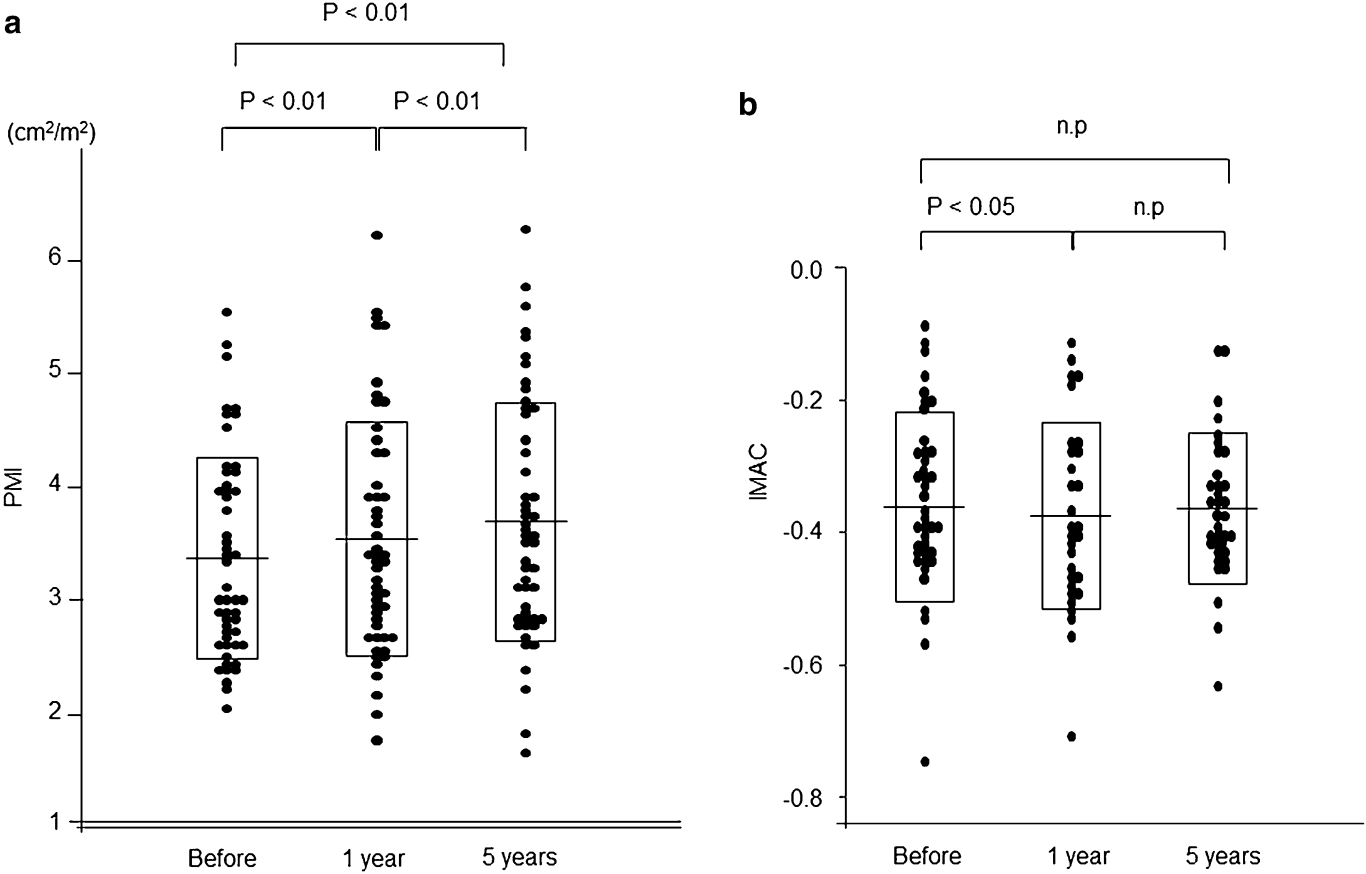
Changes in PMI and IMAC after kidney transplantation. (**a**) PMI before kidney transplantation was 3.03 cm^2^/m^2^, which significantly increased to 3.36 cm^2^/m^2^ at 1 year after kidney transplantation and 3.58 cm^2^/m^2^ at 5 years after kidney transplantation (p < 0.001 and p < 0.001, respectively, according to Dunn’s test for multiple comparisons). (**b**) IMAC significantly decreased to − 0.39 at 1 year after kidney transplantation, and no significant difference was observed after 5 years. *PMI* psoas muscle mass index, *IMAC* intramuscular adipose tissue content.

Changes in PMI and IMAC after transplantation are shown in Fig. 2. The median of PMI was 3.03 before transplantation and significantly increased to 3.36 and 3.58 at 1 and 5 years after transplantation, respectively (1 year, p < 0.001; 5 years, p < 0.001). The median of IMAC was − 0.37 before transplantation and significantly decreased to − 0.39 1 year after transplantation (p < 0.01). However, IMAC increased to − 0.36 5 years after transplantation.

### Relationship between changes in ADPN and PMI after kidney transplantation

**Figure 3:**
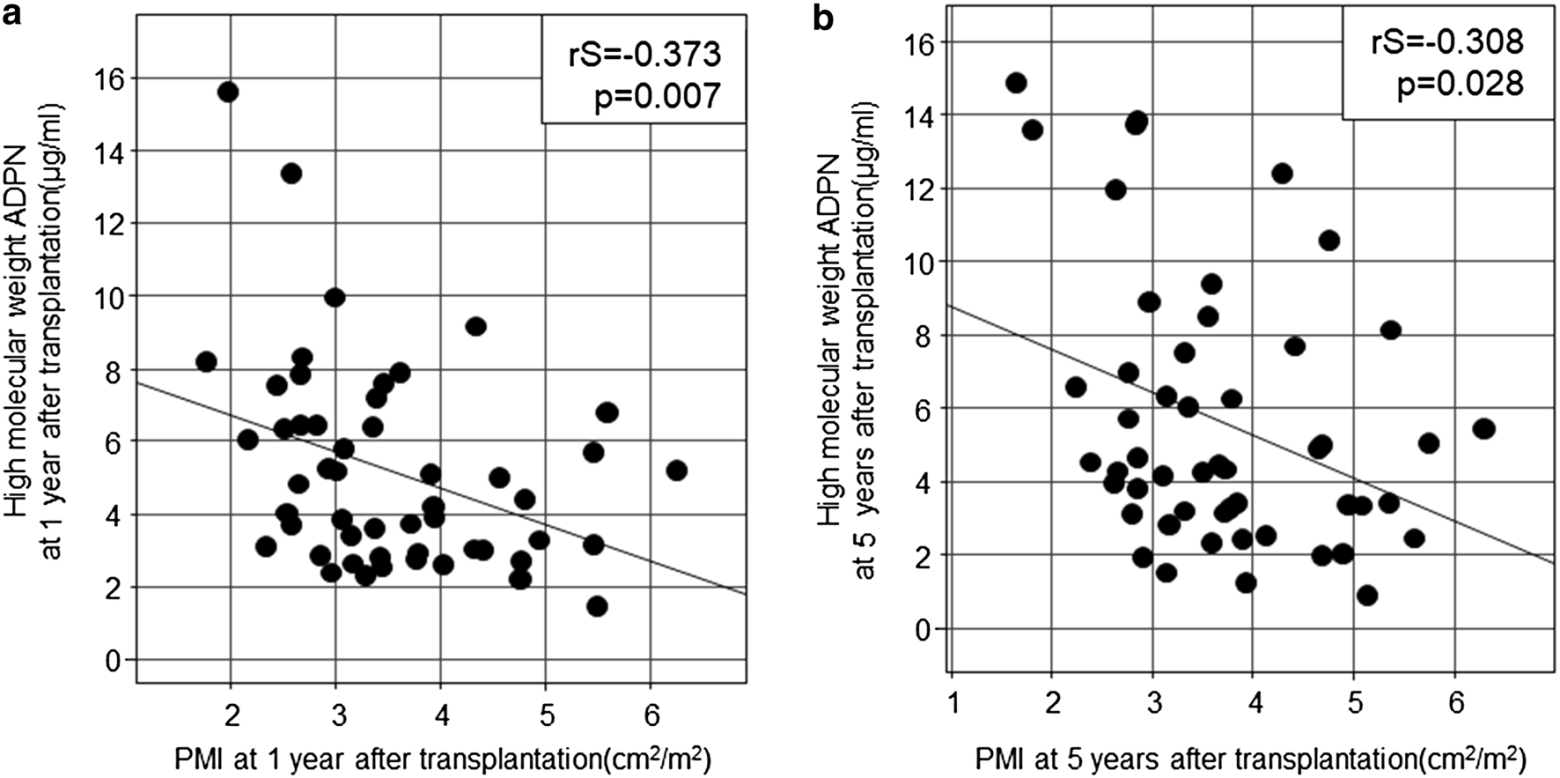
Relationship between HMW-ADPN and PMI in renal graft recipients. HMW-ADPN negatively correlated with PMI at 1 year (**a**) and 5 years (**b**) after kidney transplantation (rS = − 0.373, p = 0.007 and rS = − 0.308, p = 0.028, respectively).

HMW-ADPN, middle-molecular-weight (MMW)-ADPN and low-molecular-weight (LMW)-ADPN concentrations were 5.17 ± 2.82, 1.97 ± 0.69 and 3.73 ± 1.12 μg/mL, respectively, at 1 year after kidney transplantation, and 5.61 ± 3.61, 2.06 ± 0.82 and 3.68 ± 1.26 μg/mL, respectively, at 5 years after kidney transplantation. The serum levels of ADPN fractions were stable, showing no marked difference between 1 and 5 years after kidney transplantation. In addition, the relationships between serum HMW-ADPN concentrations and PMI at 1 and 5 years after kidney transplantation are shown in Fig. 3. HMW-ADPN negatively correlated with PMI at 1 and 5 years after kidney transplantation (rS = − 0.373, p = 0.007 and rS = − 0.308, p = 0.028, respectively). The mean change in serum HMW-ADPN levels within 4 years from 1 to 5 years after kidney transplantation negatively correlated with the mean change in PMI during the 4 years (rS = − 0.296, p = 0.034). Furthermore, multiple regression analysis of the factors causing changes in PMI identified the increase in HMW-ADPN as a factor for the decrease in PMI (Table 3). The mean change in estimated glomerular filtration rate (eGFR) within 4 years from 1 to 5 years after kidney transplantation negatively correlated with the mean change in LMW-ADPN concentrations (rS = − 0.362, p = 0.009).

**Table 3:** Factors influencing changes in PMI after kidney transplantation.

	β	SE	Stdβ	t	p
**Objective variable: Changes in PMI**					
(Constant)	0.046	0.013			
Changes in HMW-ADPN (μg/mL/year)	− 0.070	0.023	− 0.399	3.048	0.003

Explanatory variables: Sex, Age at transplantation (year), Changes in HMW-ADPN, Changes in eGFR, Changes in BMI, and Changes in HDL-C and non-HDL-C.

*PMI* psoas muscle mass index, *HMW-ADPN* high-molecular-weight adiponectin, *HDL-C* high-density lipoprotein cholesterol, *BMI* body mass index, *eGFR* estimated glomerular filtration rate.

### Relationships among the development of new onset diabetes after transplantation (NODAT), PMI and IMAC

No significant differences were observed regarding changes in PMI between non-NODAT and NODAT patients, whereas IMAC was significantly higher in NODAT patients ([3B]: p = 0.008). Multiple logistic analysis revealed that increases in IMAC and HMW-ADPN were exacerbating factors (Table 4).

**Table 4:** Factors influencing new-onset diabetes mellitus in kidney transplant subjects.

	β	SE	z	p
**Objective variable: NODAT**				
(Constant)	− 1.968	0.522		
Changes in HMW-ADPN (cm^2^/m^2^/year)	− 1.668	0.980	1.701	0.088
Changes in IMAC (/year)	76.415	35.452	2.155	0.031

Explanatory variables: Sex, Age at transplantation (year), Changes in HMW-ADPN, Changes in BMI, PMI, IMAC.

*HMW-ADPN *high–molecular-weight adiponectin, *IMAC* intramuscular adipose tissue content, *BMI* body mass index.

## Discussion

This long-term retrospective cohort study clearly showed that serum ADPN concentrations play important roles the muscle mass and fatty degeneration of muscles after kidney transplantation. Muscle mass gradually increased and the fatty degeneration of muscles was reversed in kidney transplant recipients after transplantation. Furthermore, an increase in HMW-ADPN concentrations was identified as a factor contributing to decrease in PMI. In addition, increases in PMI and IMAC were exacerbating factors for the development of NODAT.

We observed that PMI increased after kidney transplantation, and this finding indicates that sarcopenia was improved by kidney transplantation. Frailty is observed in approximately 42% of dialysis patients and has been identified as a risk factor that increases the hospitalisation rate by 1.43-fold and mortality rate by 2.6-fold, independent of age and complications^15,16^. Increases in inflammatory cytokines, an imbalance in muscle protein synthesis/degradation, reduced physical activity, an insufficient nutritional intake, metabolic acidosis, vitamin D deficiency, insulin resistance, excessive myostatin expression, increases in intramuscular angiotensin and decrease and dysfunction in satellite cells have been suggested as factors involved in the development of sarcopenia in chronic kidney disease patients^17^. Dialysis patients have a high risk of developing sarcopenia due to ageing and progressing uremic sarcopenia with prolonged dialysis. However, some of these risk factors are removed by kidney transplantation. It has been reported that 23–73% of dialysis patients suffer from malnutrition^18^. Furthermore, a decreased physical activity level is an important factor in the development of sarcopenia^19^. Patients undergoing dialysis are also at a higher risk of sarcopenia due to the loss of proteins and amino acids into the dialysis solution^20^. Increased muscle mass following kidney transplantation may be attributed to an increased level of physical activity, as well as increased protein intake without any loss of proteins that would otherwise occur during dialysis. We herein objectively demonstrated that PMI, which reflects muscle mass, clearly increased, and IMAC, which reflects muscle quality, was maintained for a long time after kidney transplantation.

Fat cells that have the ability to secrete hormones secrete anti-inflammatory cytokines, such as ADPN and leptin, as well as pro-inflammatory cytokines such as PAI-1, IL-6, and TNF-α. Among these cytokines, TNF-α and IL-6 are known to promote sarcopenia^21^. Studies also suggest that with aging, the serum ADPN concentration increases from adulthood while skeletal muscle mass peaks around adulthood and decreases gradually^22^. Previous studies also demonstrated that ADPN and AdipoR1 regulate the expression and activation of PPARγcoactivator-1α [PCG-1α], a key component of sarcopenia, by Ca^2+^ signalling, AMP-activated protein kinase (AMPK), and SIRT1. Thus, there is an increasing interest in the role of ADPN and AdipoR1 in the regulation of skeletal muscle fibers^23^. We also revealed that an increase in HMW-ADPN concentrations was identified as a factor contributing to decrease in PMI. In the present study, a negative correlation was observed between serum ADPN concentrations and muscle mass, a marker of sarcopenia. Although this finding seems to conflict with previous reports, similar observation was also reported. There was a significant inverse correlation between serum adiponectin levels with arm lean mass and muscle strength in patients with HF^24^. In addition, muscle weakness in the limbs was reported to be related to an increase in serum ADPN concentrations in elderly patients and leg muscle strength inversely correlated with blood ADPN concentrations in patients aged ≥ 70 years^25^. A previous study confirmed that the percentage of type IIB muscle fibres increased in ADPN knockout mice^26^, and an epidemiological study on 461 elderly subjects showed that IIB fibres were reduced in those with high blood ADPN concentrations^27^. In addition, a recent study showed that administration of AdipoRon, an adiponectin receptor agonist, increases blood ADPN concentrations and induces atrophy of type II muscle fibres via the adiponectin receptor 1-AMP-dependent protein kinase signal pathway in mice^28^. Since the present study found a negative correlation between blood levels of HMW-ADPN and the cross-sectional area of the greater psoas muscle, the muscle fibres that correlated with ADPN may be primarily type II muscle fibres. Collectively, these findings and the present results suggest that muscles of kidney transplant patients would similarly react to HMW-ADPN as aged cases. HMW-ADPN exerts different effects on different muscle fibre types, i.e. HMW-ADPN increases the percentage of type I fibres but decreases that of type II fibres, as the mechanism of action of ADPN in sarcopenia.

Our study showed that increases in IMAC were exacerbating factors for the development of NODAT. Since the mass of muscle, a target organ of insulin, decreases in sarcopenia, patients with sarcopenia have insulin resistance. In obese patients with sarcopenia (sarcopenic obesity), blood levels of IL-6 and CRP are elevated, and these factors lead to an increase in insulin resistance and decrease in skeletal muscle mass^29, 30^. This would be a vicious cycle in sarcopenic obesity. Moreover, insulin also stimulates protein synthesis. Therefore, skeletal muscle synthesis may be suppressed in diabetic patients, and leg neuropathy has been strongly implicated in muscle weakness^31^. Landi et al. reported that sarcopenia and diabetes may be interrelated via factors such as insulin resistance, inflammatory cytokines and mitochondrial hypofunction^32^. Furthermore, in addition to subcutaneous and visceral fat, ectopic fat, called the third fat, is now being investigated. Akima et al. showed that the accumulation of fat in the muscles of elderly individuals was related to sarcopenia and impaired motor function^33^, and a decline in mitochondrial function and fat accumulation in muscles has been suggested to increase insulin resistance^34^. Although the state of uremic sarcopenia is ameliorated and muscle mass increases after kidney transplantation, serial increases in IMAC, i.e. the progression of the fatty degeneration of muscles, are considered to be involved in the development of NODAT. Therefore, the preservation of not only muscle mass, but also muscle quality are important for the prevention of NODAT.

The limitations of the present study include (1) the small number of subjects, (2) its retrospective design, and (3) evaluation limited to muscle mass of the greater psoas and multifidus muscles. Further studies are warranted to examine other muscles, such as the extensor digitorum longus muscle, which is reported to have a high content of type II muscle fibres and to investigate differences among muscles in the same individuals. We also intend to perform a prospective study, extend the observation period and examine relationships with the prognoses of and cardiovascular complications in these patients.

## Conclusion

Our study showed that PMI gradually increased and IMAC decreased after kidney transplantation. The increase in PMI was associated with HMW-ADPN levels after kidney transplantation, which indicates its involvement in the development of NODAT in renal allograft recipients. Therefore, sufficient exercise therapy is also considered to be necessary after transplantation to preserve skeletal muscle mass and prevent the fatty degeneration of muscle.

## Methods

### Patients

Fifty-one patients who underwent kidney transplantation since 1998 at our hospital and showed stable renal function for at least 6 months thereafter (40 recipients from living donors and 11 recipients from deceased donors; 31 males and 20 females) were retrospectively examined. The causes of renal failure in the transplant patients were chronic glomerulonephritis (n = 35), purpura nephritis (n = 3), membranoproliferative glomerulonephritis (n = 3), hypoplasia (n = 3), nephrosclerosis (n = 3), polycystic kidney disease (n = 2), focal segmental glomerulosclerosis (n = 1), reflux nephropathy (n = 1). Since the time required for the stabilisation of kidney graft functions differs between kidney transplant recipients from living donors and those from deceased donors, the changes in serum ADPN fractions within 4 years from 1 to 5 years after transplantation were investigated in the present study.

The relationship between skeletal muscle masses evaluated by PMI 1 and 5 years after transplantation and the serum levels of ADPN fractions (HMW, MMW and LMW fractions) were assessed as the primary endpoint. The relationship between intramuscular adipose tissue contents (IMAC) at 1 and 5 years after transplantation, the serum levels of ADPN fractions and changes in PMI and IMAC after transplantation and among NODAT, PMI, IMAC and serum ADPN fractions were investigated as the secondary endpoints.

The following clinical factors were evaluated: age at transplantation, gender, duration of dialysis, blood pressure at 1 and 5 years after transplantation, BMI, serum creatinine (SCr), eGFR, serum albumin, T chol, HDL-C, non-HDL-C (T chol-HDL-C), ADPN fractions (HMW, MMW and LMW), types of immunosuppressants used, statins and anti-hypertensive agents.

The present study did not include any vulnerable populations, such as prisoners, subjects with reduced mental capacity due to illness or age or children. Furthermore, we used blood samples, radiological scans and renal biopsy specimens. The study protocol was approved by the Ethics Committee of Kanazawa Medical University (Kanazawa Medical University Epidemiological Study Review No. I293). All patients provided written informed consent, and the study was conducted according to the principles of the Declaration of Helsinki and Istanbul.

### Measurement methods

Serum levels of total HMW-, MMW- and LMW-ADPN were measured using a sensitive enzyme-linked immunosorbent assay kit (SEKISUI MEDICAL Co., Tokyo, Japan). Renal function was evaluated based on eGFR [194 × SCr^−1.094 ^× age^−0.287^ (× 0.739 for females), mL/min/1.73 m^2^]. eGFR was calculated based on patient SCr levels, as described previously^35^.

### Imaging analysis

The skeletal muscle mass index proposed by Prado et al., calculated by dividing total skeletal muscle mass at the L3 level by the square of height, is a typical index for evaluating skeletal muscle mass by CT^36^. Evaluation method of CT image are shown in SI Fig. S1. we calculated PMI (cm^2^/m^2^) as the cross-sectional area of the greater psoas muscle (cm^2^)/height^2^ (m^2^) using image analysis software (MITANI Co., Ltd., Fukui, Japan) and examined regions of interest (ROI) in the bilateral greater psoas muscles in a slice of abdominal CT at the L3 level. IMAC was measured using AZE Virtual Place (AZE Co., Ltd., Kanagawa, Japan) and setting ROIs over the bilateral multifidus muscles in a slice of abdominal CT at the L3 level. Moreover, ROIs were set in 4 areas of subcutaneous fat distant from the large vessels, and the mean CT value of the multifidus muscle/mean CT value of the subcutaneous fat region in the back was calculated and evaluated.

### Statistical analyses

All continuous variables are expressed as median values and interquartile ranges. Comparisons between sexes were made using the Mann–Whitney test. The relationships between PMI and IMAC before transplantation, duration of dialysis and age at transplantation as well as between the mean change in eGFR and mean change in serum ADPN levels were examined according to Spearman’s rank correlation coefficient. Changes in PMI and IMAC after transplantation were evaluated using Dunn’s test. Factors affecting PMI and IMAC before transplantation and those affecting mean changes in serum HMW-ADPN levels and PMI were analysed using multiple regression analysis. In addition, factors that affected the development of posttransplant diabetes mellitus were assessed by multiple logistic regression analysis. The statistical software Stat Flex Ver6 (Artech Co., Ltd., Osaka, Japan) was used for analyses.

## Supplementary information

Supplementary Figure 1

## Data Availability

The datasets analysed during the current study are available from the corresponding author on reasonable request.
