# Supplementary material for: Long-term retrospective observation study to evaluate effects of adiponectin on skeletal muscle in renal transplant recipients
Source: Sci Rep. 2020 Jul 1;10:10723. doi: 10.1038/s41598-020-67711-1 (PMC7330033; doi:10.1038/s41598-020-67711-1)

Long-term retrospective observation study to evaluate effects of adiponectin on skeletal muscle in renal transplant recipients

Hiroki Adachi, MD, PhD; Keiji Fujimoto, MD, PhD; Ai Fujii, MD; Keita Yamasaki, MD; Keiichiro Okada, MD, PhD; Toshikazu Matsuura, MD; Kazuaki Okino, MD, PhD; Kengo Furuichi, MD, PhD; Hitoshi Yokoyama, MD, PhD*

Department of Nephrology, Kanazawa Medical University School of Medicine, 1-1 Daigaku, Uchinada, Ishikawa 920-0293, Japan

*Corresponding author:

Professor, Hitoshi Yokoyama, MD, PhD

Department of Nephrology

Kanazawa Medical University School of Medicine

Daigaku, Uchinada, Ishikawa 920-0293, Japan

Telephone No.: +81-76-218-8166

Fax No.: +81-76-286-2786

E-mail address: h-yoko@kanazawa-med.ac.jp


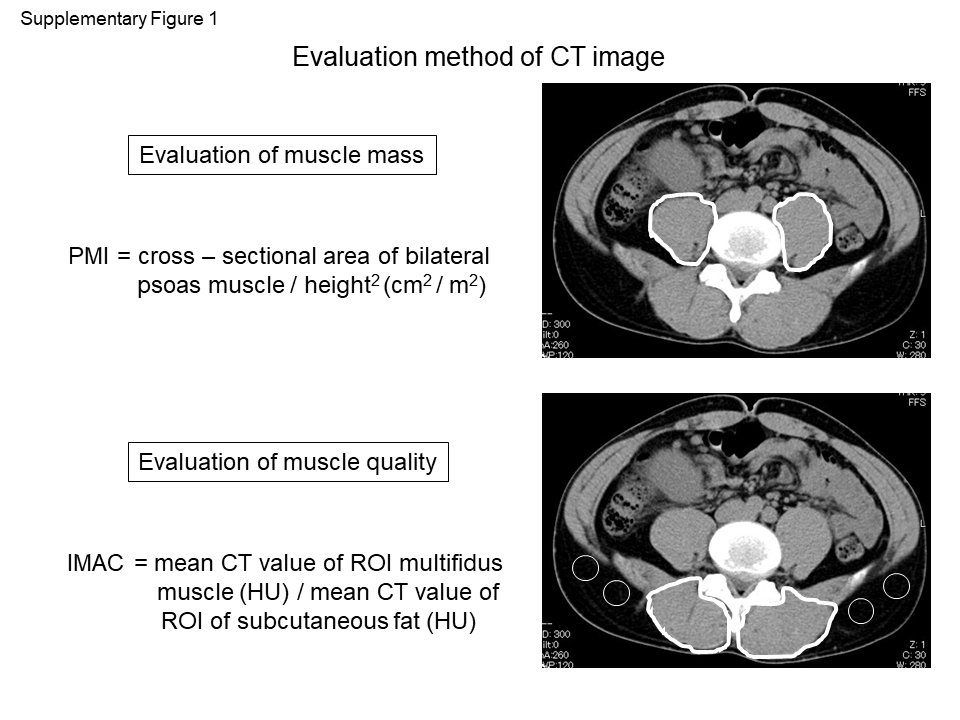

Supplement: Supplementary file 1 — Supplementary Figure 1 [file 41598_2020_67711_MOESM1_ESM.docx]
